# Association between pediatric asthma and adult polycystic ovarian syndrome (PCOS): a cross-sectional analysis of the UAE healthy future Study (UAEHFS)

**DOI:** 10.3389/fendo.2023.1022272

**Published:** 2023-05-24

**Authors:** Nirmin F. Juber, Abdishakur Abdulle, Abdulla AlJunaibi, Abdulla AlNaeemi, Amar Ahmad, Andrea Leinberger-Jabari, Ayesha S. Al Dhaheri, Eiman AlZaabi, Fatima Mezhal, Fatma Al-Maskari, Fatme Alanouti, Habiba Alsafar, Juma Alkaabi, Laila Abdel Wareth, Mai Aljaber, Marina Kazim, Michael Weitzman, Mohammed Al-Houqani, Mohammed Hag-Ali, Naima Oumeziane, Omar El-Shahawy, Scott Sherman, Syed M. Shah, Tom Loney, Wael Almahmeed, Youssef Idaghdour, Raghib Ali

**Affiliations:** ^1^ Public Health Research Center, New York University Abu Dhabi, Abu Dhabi, United Arab Emirates; ^2^ Department of Pediatrics, Zayed Military Hospital, Abu Dhabi, United Arab Emirates; ^3^ Department of Cardiology, Zayed Military Hospital, Abu Dhabi, United Arab Emirates; ^4^ Department of Nutrition and Health, College of Medicine and Health Sciences; United Arab Emirates University, Al-Ain, United Arab Emirates; ^5^ Department of Pathology, Sheikh Shakhbout Medical City, Abu Dhabi, United Arab Emirates; ^6^ Institute of Public Health, College of Medicine and Health Sciences, United Arab Emirates University, Al-Ain, United Arab Emirates; ^7^ Zayed Center for Health Sciences, United Arab Emirates University, Al-Ain, United Arab Emirates; ^8^ College of Natural and Health Sciences, Zayed University, Abu Dhabi, United Arab Emirates; ^9^ Center for Biotechnology, Khalifa University of Science and Technology, Abu Dhabi, United Arab Emirates; ^10^ Department of Genetics and Molecular Biology, Khalifa University of Science and Technology, Abu Dhabi, United Arab Emirates; ^11^ Department of Biomedical Engineering, Khalifa University of Science and Technology, Abu Dhabi, United Arab Emirates; ^12^ Department of Internal Medicine, College of Medicine and Health Sciences, United Arab Emirates University, Al-Ain, United Arab Emirates; ^13^ Department of Laboratory Medicine, National Reference Laboratory, Abu Dhabi, United Arab Emirates; ^14^ Healthpoint Hospital, Abu Dhabi, United Arab Emirates; ^15^ Abu Dhabi Blood Bank Services, SEHA, Abu Dhabi & Al-Ain, United Arab Emirates; ^16^ Department of Environmental Medicine, New York University of Medicine, New York, United States; ^17^ Department of Medicine, College of Medicine and Health Sciences, United Arab Emirates University, Al-Ain, United Arab Emirates; ^18^ Faculty of Health Sciences, Higher Colleges of Technology, Abu Dhabi, United Arab Emirates; ^19^ Department of Population Health, New York University School of Medicine, New York, United States; ^20^ College of Medicine, Mohammed Bin Rashid University of Medicine and Health Sciences, Dubai, United Arab Emirates; ^21^ Heart and Vascular Institute, Cleveland Clinic Abu Dhabi, Abu Dhabi, United Arab Emirates; ^22^ Medical Research Council (MRC) Epidemiology Unit, University of Cambridge, Cambridge, United Kingdom

**Keywords:** asthma, pediatric asthma, polycystic ovarian syndrome, PCOS, epidemiology, risk factors, public health

## Abstract

**Introduction:**

Asthma and polycystic ovarian syndrome (PCOS) are linked in several possible ways. To date, there has been no study evaluating whether pediatric asthma is an independent risk factor for adult PCOS. Our study aimed to examine the association between pediatric asthma (diagnosed at 0-19 years) and adult PCOS (diagnosed at ≥20 years). We further assessed whether the aforementioned association differed in two phenotypes of adult PCOS which were diagnosed at 20-25 years (young adult PCOS), and at >25 years (older adult PCOS). We also evaluated whether the age of asthma diagnosis (0-10 vs 11-19 years) modified the association between pediatric asthma and adult PCOS.

**Material and methods:**

This is a retrospective cross-sectional analysis using the United Arab Emirates Healthy Future Study (UAEHFS) collected from February 2016 to April 2022 involving 1334 Emirati females aged 18-49 years. We fitted a Poisson regression model to estimate the risk ratio (RR) and its 95% confidence interval (95% CI) to assess the association between pediatric asthma and adult PCOS adjusting for age, urbanicity at birth, and parental smoking at birth.

**Results:**

After adjusting for confounding factors and comparing to non-asthmatic counterparts, we found that females with pediatric asthma had a statistically significant association with adult PCOS diagnosed at ≥20 years (RR=1.56, 95% CI: 1.02-2.41), with a stronger magnitude of the association found in the older adult PCOS phenotype diagnosed at >25 years (RR=2.06, 95% CI: 1.16-3.65). Further, we also found females reported thinner childhood body size had a two-fold to three-fold increased risk of adult PCOS diagnosed at ≥20 years in main analysis and stratified analyses by age of asthma and PCOS diagnoses (RR=2.06, 95% CI: 1.08-3.93 in main analysis; RR=2.74, 95% CI: 1.22-6.15 among those diagnosed with PCOS > 25 years; and RR=3.50, 95% CI: 1.38-8.43 among those diagnosed with asthma at 11-19 years).

**Conclusions:**

Pediatric asthma was found to be an independent risk factor for adult PCOS. More targeted surveillance for those at risk of adult PCOS among pediatric asthmatics may prevent or delay PCOS in this at-risk group. Future studies with robust longitudinal designs aimed to elucidate the exact mechanism between pediatric asthma and PCOS are warranted.

## 1 Introduction

Asthma is a multifactorial respiratory disease defined by reversible airway hyperactivity and a wide range of symptoms (1). Inflammation is a key factor in the pathology of asthma, and cross-communication between the airways and inflammatory mediators leads to inflammation that is not only confined to the local airways but also tends to be systemic (2). Asthma is a common pediatric disease with more than 80% of first asthma episodes happening in the first six years of life (3). The main risk factors associated with pediatric asthma include genetic predisposition, viral respiratory infections, and female sex hormones (4). In addition, pediatric asthma has long-term health consequences and is known to be associated with adult non-communicable diseases such as hypertension and diabetes (5).

Polycystic Ovarian Syndrome (PCOS) is a complex endocrine disorder affecting 5-10% of females of reproductive age (6). PCOS is a multifactorial disorder and risk factors associated with PCOS include genetic predisposition, hormonal factors, as well as maternal environmental factors (e.g. metabolic disturbance during pregnancy) (7, 8). Previous studies found that females with PCOS have an increased risk of developing subsequent metabolic disorders, such as cardiovascular disease, hypertension, and diabetes (7, 9, 10). The etiology of PCOS is not exactly known, however, chronic systemic inflammation has been proposed as one of the possible mechanisms (8, 11). In addition, PCOS may have its early-life origins through exposure to excess androgens at any stage from fetal development to childhood period (7, 8).

Asthma and PCOS are linked in several possible ways. Previous studies have established the association between PCOS and subsequent asthma among reproductive-aged females (12–14). There is clinical overlap between asthma and PCOS (15), including alterations in gut microbiota (16–18), menstrual cycle abnormalities (19–21), infertility (22, 23), obesity (24–26), and insulin resistance that was found in asthmatics as well as females with PCOS (27, 28). Previous epidemiological studies have also found an association between PCOS and subsequent asthma (13, 29). However, there is a limited epidemiological study on another possible direction of the association between asthma and subsequent PCOS, including our previous work examining the association between asthma diagnosed at <25 years with subsequent PCOS diagnosed at ≥25 years (30). Asthma may be associated with subsequent PCOS as asthma and PCOS are multifactorial complex diseases and they shared pathophysiological mechanisms, including female hormonal disturbance, systemic/low-grade inflammation, as well as obesity and, metabolic syndrome (11–14, 31). We thus predict that pediatric asthma might be associated with adult PCOS, independent of other relevant risk factors found in our dataset.

Our study aimed to examine the association between pediatric asthma (diagnosed at 0-19 years of age) and adult PCOS (overall adult PCOS: diagnosed at ≥20 years, young adult PCOS: diagnosed at 20-25 years, and older adult PCOS: diagnosed at 25-49 years). We also assessed whether the age of asthma diagnosis (childhood asthma: diagnosed at 0-10 years, and adolescent asthma: diagnosed at 11-19 years) modified the association between pediatric asthma and adult PCOS. Finally, in the main analysis and stratified models, we performed restriction analysis by body size at 10 years old and health status up to 10 years old, to better address the potential mediating effect of childhood body size and childhood health on the association between pediatric asthma and adult PCOS.

## 2 Material and methods

### 2.1 Study design, participants, and setting

**Figure 1:**
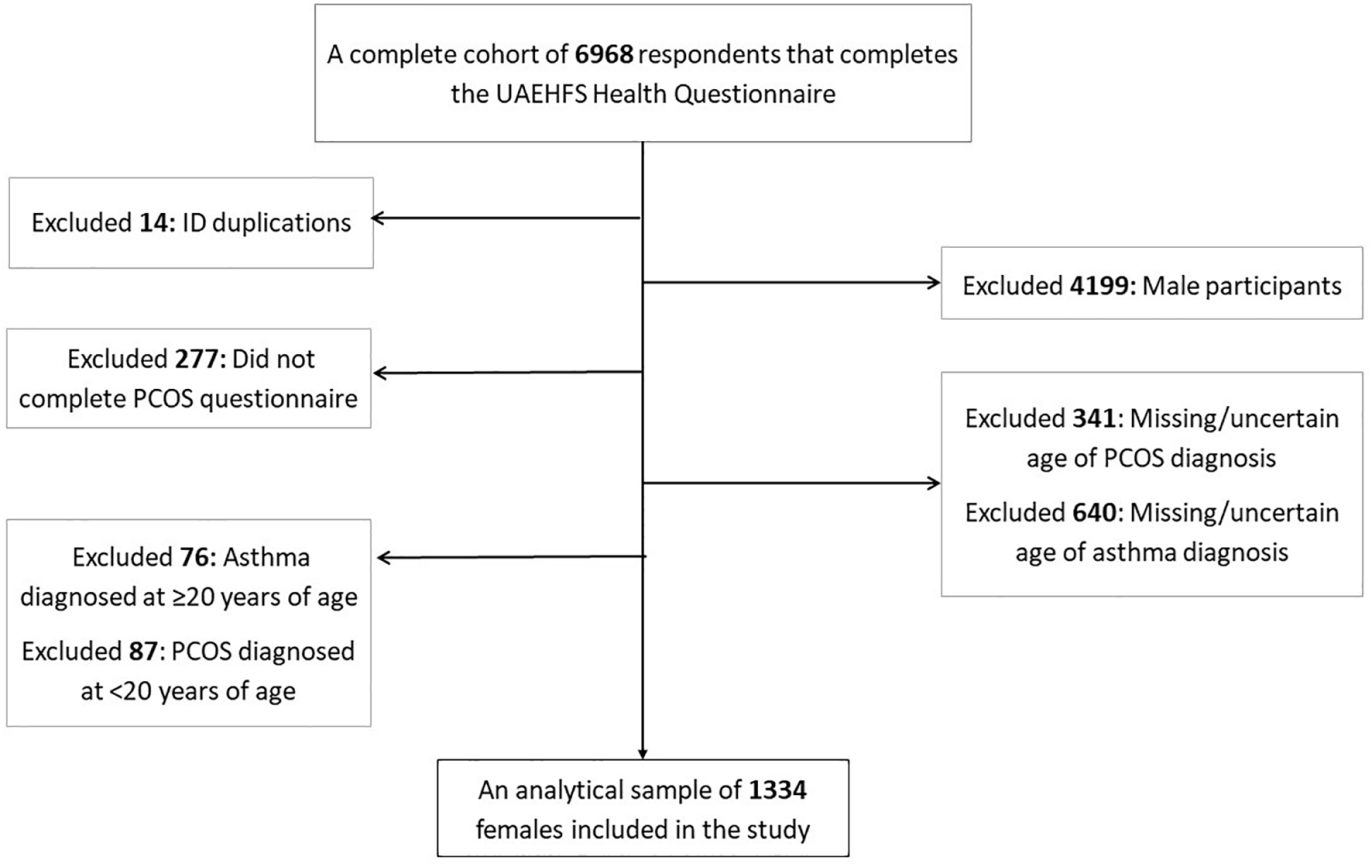
Flowchart of the final analytical sample included in the study.

This is a retrospective cross-sectional study using the United Arab Emirates Healthy Future Study (UAEHFS) collected from February 2016 to April 2022. We included all 1334 females aged 18-49 years who had complete information on the age of asthma and PCOS diagnosis (**Figure 1**). The study design, questionnaire, and methodologies of the UAEHFS are described elsewhere (32). In brief, the UAEHFS is an ongoing population-based prospective cohort study among Emirati nationals aged 18 years or above. A convenience sample of Emirati individuals was invited to participate from across the UAE. Multiple recruitment centers were set up across the country where participants filled out the questionnaire and had some physical measurements. Due to the COVID-19 pandemic, the recruitment shifted to online-based starting in April 2020, and an online questionnaire was introduced to the new participants. Physical measurements, such as body mass index (BMI), were taken in the participating centers for new participants that filled out and returned the online questionnaire.

### 2.2 Ethical approval

The study and its procedures have been reviewed and approved by the Institutional Review Board at New York University Abu Dhabi, Dubai Health Authority, Ministry of Health and Prevention in the UAE, and Health Research and Technology Committee, reference number DOH/HQD/2020/516. Written consent was obtained from participants at the centers or by filling out an online consent form before data collection started.

### 2.3 Measurements

We analyzed self-reported physician diagnoses for asthma based on the questionnaire response to: “Has a doctor ever told you that you had asthma?”. The age at asthma diagnosis was extracted from the questionnaire response to: “What was your age when asthma was first diagnosed?”, and pediatric asthma was defined as asthma first diagnosed at 19 years of age or younger (pediatric population) (5, 33). Similarly, self-reported physician diagnosis for PCOS was used based on the questionnaire response to: “Has a doctor ever told you that you had PCOS?”. The age at PCOS diagnosis was extracted from the questionnaire response to: “How old were you when the doctor first told you that you had PCOS?”, and adult PCOS was defined as PCOS first diagnosed at 20 years of age or above (34). Overall health up to 10 years old was determined based on the questionnaire response to: “In general, how was your health in childhood (less than 10 years old)?”, we then categorized childhood health status variable into two categories: poor or fair and good or excellent based on the responses. Body size at 10 years old was determined based on the questionnaire responses to: “When you were 10 years old, compared to average, would you describe yourself as: about average, thinner, or plumper?”, we then classified body size at 10 years old into four categories of around average, average, thinner, and plumper. Age was constructed based on the questionnaire response to “What is your date of birth”, and we kept its continuous form in our analysis. Urbanicity at birth and current urbanicity were determined based on the questionnaire response to: “Where do you and your family live around the time of your birth?”, and “Where do you and your family live now?”, respectively. We then categorized the city as an urban area and non-cities (villages, deserts, islands, and others) as rural or other non-urban. Parental smoking (at birth) was constructed based on the questionnaire response to “Did your mother or father smoke regularly around the time when you were born?” (no/yes). Parental education and education attainment were constructed based on the questionnaire response to: “What level of education did your father or mother complete?”, and “What is the highest level of education that you have completed?”, respectively. We categorized education levels into three categories (≤6 years, >6 to 12 years, and 12+ years of schooling). Birthweight which was determined based on the questionnaire item: “What was your birth weight (in kg)?”, was categorized later into low birthweight (<2.5 kg) and normal birthweight (2.5+ kg). BMI was calculated using the Tanita MC 780 by nurses at the recruitment centers, and the values for each participant were recorded and its continuous form was used in our analysis.

### 2.4 Statistical analysis

Characteristics of the study participants were evaluated using numbers with percentage (n, %) for each categorical variable and mean with standard deviation (mean ± SD) for each continuous variable (**Table 1**). To establish asthma as an exposure preceding PCOS as an outcome, we relied on age at disease diagnosis and excluded those with adult asthma (diagnosed at >19 years of age) and adolescent PCOS (diagnosed at <20 years of age). We then fitted a Poisson regression model with robust variance to estimate the risk ratio (RR) and its 95% confidence interval (95% CI) to assess the association between pediatric asthma (diagnosed at 0-19 years of age) and adult PCOS (diagnosed at ≥20 years of age) (**Tables 2**–**4**) (35). We examined the RR and its 95% CI in the crude and adjusted models, adjusting for age (5, 36), urbanicity at birth (37, 38), and parental smoking (37, 39). Due to a high amount of missing values (>50%) on parental education (40, 41) and birthweight (42, 43), we excluded these potential confounding factors in the regression analysis even though these are important confounding factors in this study. In addition, the missing indicator method was used to handle uncertain values from missing, prefer not to answer (PFA), and do not know (DN) responses. Analyses were carried out using STATA 17.0 (StataCorp, TX). *P* values <0.05 were considered statistically significant.

**Table 1:** Characteristics of female study participants based on pediatric asthma status, missing or uncertain vales are not shown (N=1334).

Characteristics	Non-asthmatics (N= 1204)	With pediatric asthma (N= 130)
Age, year (mean ± SD)	23.8 ± 6.1	24.8 ± 6.4
BMI, kg/m2 (mean ± SD)	23.2 ± 4.7	28.5 ± 7.6
Marital status, n (%)		
Single	914 (75.9)	100 (76.9)
Married	235 (19.5)	26 (20.0)
Divorced or separated	38 (3.2)	4 (3.1)
Widow or widower	17 (1.4)	0 (0)
Urbanicity at questionnaire, n (%)		
Rural or other non-urbans	173 (14.4)	14 (10.8)
Urban (city)	1002 (83.2)	116 (89.2)
Urbanicity at birth, n (%)		
Rural or other non-urbans	233 (19.4)	21 (16.2)
Urban (city)	899 (74.6)	102 (78.5)
Education attainment, n (%)		
6 years of schooling or below	48 (4.0)	2 (1.5)
>6-12 years of schooling	577 (47.9)	58 (44.6)
>12 years of schooling	579 (48.1)	70 (53.9)
Parental education attainment, n (%)		
6 years of schooling or below	51 (4.3)	5 (3.9)
>6-12 years of schooling	150 (12.5)	18 (13.9)
>12 years of schooling	183 (15.2)	28 (21.5)
Overall health up to 10 years, n (%)		
Poor or fair	132 (11.0)	33 (25.4)
Good or excellent	1072 (89.0)	97 (74.6)
Body size at 10 years, n (%)		
About average	436 (36.2)	54 (41.5)
Thinner	518 (43.0)	46 (35.4)
Plumper	101 (8.4)	21 (16.2)
Parental smoking at birth, n (%)		
No	819 (68.0)	82 (63.1)
Yes	270 (22.4)	38 (29.2)
Birthweight, n (%)		
<2.5 kg	189 (15.7)	34 (26.2)
2.5+ kg	288 (23.9)	40 (30.8)
With PCOS diagnosed at> 20 years, n (%)		
No	1102 (91.5)	111 (85.4)
Yes	102 (8.5)	19 (14.6)

**Table 2:** Modified Poisson regression analysis between females with pediatric asthma history (diagnosed at 0-19 years of age) and adult PCOS (diagnosed at ≥20 years of age), in total population and restricted by potential mediators (N=1334).

	Crude Model			Adjusted Model^a^		
	RR	[95% CI]	*P*	RR	[95% CI]	*P*
Main analysis						
*Non-asthmatics (N=1204)*	(Reference)			(Reference)		
*Pediatric asthmatics (N=130)*	1.73	1.10-2.72	0.019	1.56	1.02 -2.41	0.040
Restriction analysis by body size at 10 years old						
About average						
*Non-asthmatics (N= 436)*	(Reference)			(Reference)		
*Pediatric asthmatics (N= 54)*	1.66	0.78-3.57	0.192	1.39	0.73 -2.66	0.323
Thinner						
*Non-asthmatics (N=518)*	(Reference)			(Reference)		
*Pediatric asthmatics (N=46)*	2.00	1.01-3.99	0.048	2.06	1.08-3.93	0.028
Plumper						
*Non-asthmatics (N=101)*	(Reference)			(Reference)		
*Pediatric asthmatics (N=21)*	1.03	0.32-3.29	0.959	1.13	0.27 -4.69	0.871
Restriction analysis by childhood health status up to 10 years old						
Poor or fair						
*Non-asthmatics (N=132)*	(Reference)			(Reference)		
*Pediatric asthmatics (N=33)*	2.00	0.73-5.47	0.177	2.41	0.95 -6.10	0.063
Excellent or good						
*Non-asthmatics (N=1072)*	(Reference)			(Reference)		
*Pediatric asthmatics (N=97)*	1.68	0.99-2.84	*0.051*	1.44	0.88-2.38	0.149

a Adjusted for age (continuous), urbanicity at birth (rural/urban), and parental smoking at birth (no/yes).

**Table 3:** Modified Poisson regression analysis between females with pediatric asthma and adult PCOS, stratified by the age of PCOS diagnosis (20-25 years vs >25 years), in total population and restricted by potential mediators (N=1334).

	Age of PCOS diagnosis: 20-25 years of age						Age of PCOS diagnosis: > 25 years of age					
	Crude Model			Adjusted Model^a^			Crude Model			Adjusted Model^a^		
	RR	[95% CI]	*P*	RR	[95% CI]	*P*	RR	[95% CI]	*P*	RR	[95% CI]	*P*
Main analysis												
*Non-asthmatics*	(Reference)			(Reference)			(Reference)			(Reference)		
*Pediatric asthmatics*	1.32	0.65 -2.70	0.443	1.33	0.65 -2.72	0.442	2.40	1.27 -4.53	0.007	2.06	1.16 -3.65	0.013
Restriction analysis by body size at 10 years old												
About average												
*Non-asthmatics*	(Reference)			(Reference)			(Reference)			(Reference)		
*Pediatric asthmatics*	0.40	0.06-2.92	0.367	0.39	0.06-2.79	0.350	3.91	1.53-9.98	0.004	3.40	1.49-7.73	0.004
Thinner												
*Non-asthmatics*	(Reference)			(Reference)			(Reference)			(Reference)		
*Pediatric asthmatics*	1.46	0.46-4.63	0.523	1.55	0.49-4.94	0.454	2.87	1.13-7.26	0.026	2.74	1.22-6.15	0.015
Plumper												
*Non-asthmatics*	(Reference)			(Reference)			(Reference)			(Reference)		
*Pediatric asthmatics*	1.92	0.54-6.85	0.316	2.55	0.65-9.96	0.180	N/A			N/A		
Restriction analysis by childhood health status up to 10 years old												
Poor or fair												
*Non-asthmatics*	(Reference)			(Reference)			(Reference)			(Reference)		
*Pediatric asthmatics*	1.42	0.30-6.73	0.657	1.63	0.37-7.10	0.517	3.05	0.72-13.0	0.132	3.11	0.61-15.7	0.170
Excellent or good												
*Non-asthmatics*	(Reference)			(Reference)			(Reference)			(Reference)		
*Pediatric asthmatics*	1.31	0.58-2.97	0.512	1.25	0.55-2.82	0.599	2.30	1.11-4.77	0.026	1.87	0.94-3.69	0.073

a Adjusted for age (continuous), urbanicity at birth (rural/urban), and parental smoking at birth (no/yes). N/A reflects insufficient sample size.

**Table 4:** Modified Poisson regression analysis between females with pediatric asthma and adult PCOS, stratified by the age of asthma diagnosis (0-10 vs >11-19 years), in total population and restricted by potential mediators (N=1334).

	Age of asthma diagnosis: 0-10 years						Age of asthma diagnosis: 11-19 years					
	Crude Model			Adjusted Model^a^			Crude Model			Adjusted Model^a^		
	RR	[95% CI]	*P*	RR	[95% CI]	*P*	RR	[95% CI]	*P*	RR	[95% CI]	*P*
Main analysis												
*Non-asthmatics*	(Reference)			(Reference)			(Reference)			(Reference)		
*Pediatric asthmatics*	1.59	0.94-2.68	0.082	1.46	0.89-2.39	0.130	2.27	1.01-5.10	0.047	1.99	0.93-4.23	0.075
Restriction analysis by body size at 10 years old												
About average												
*Non-asthmatics*	(Reference)			(Reference)			(Reference)			(Reference)		
*Pediatric asthmatics*	1.46	0.60-3.54	0.405	1.18	0.56-2.49	0.657	2.57	0.71-9.25	0.150	2.39	0.80-7.19	0.120
Thinner												
*Non-asthmatics*	(Reference)			(Reference)			(Reference)			(Reference)		
*Pediatric asthmatics*	1.69	0.72-4.00	0.229	1.68	0.76-374	0.202	2.88	1.04-7.98	0.042	3.50	1.38-8.43	0.008
Plumper												
*Non-asthmatics*	(Reference)			(Reference)			(Reference)			(Reference)		
*Pediatric asthmatics*	1.20	0.38-3.78	0.753	1.36	0.36-5.17	0.649	N/A			N/A		
Restriction analysis by childhood health status up to 10 years old												
Poor or fair												
*Non-asthmatics*	(Reference)			(Reference)			(Reference)			(Reference)		
*Pediatric asthmatics*	2.28	0.83-6.18	0.106	2.54	1.03-6.30	0.044	N/A			N/A		
Excellent or good												
*Non-asthmatics*	(Reference)			(Reference)			(Reference)			(Reference)		
*Pediatric asthmatics*	1.39	0.74-2.66	0.307	1.23	0.66-2.28	0.511	2.65	1.20-5.87	0.016	2.13	1.01-4.50	0.047

a Adjusted for age (continuous), urbanicity at birth (rural/urban), and parental smoking at birth (no/yes). N/A reflects insufficient sample size.

## 3 Results

**Table 1** summarizes the descriptive characteristics of study participants based on their pediatric asthma status. Out of 1334 females aged 18 to 49 years, 130 females reported ever being diagnosed with asthma when they were 0-19 years of age (pediatric asthma prevalence = 9.75%). Compared to non-asthmatics, females with pediatric asthma were older (24.8 ± 6.4 vs 23.8 ± 6.1 years), had a higher BMI (28.5 ± 7.6 vs 23.2 ± 4.7 kg/m^2^), a greater proportion resided in urban areas (89.2% vs 83.2%), a greater proportion had >12 years of schooling (high level of education) (53.9% vs 48.1%), and a higher proportion diagnosed with PCOS (14.6% vs 8.5%). In terms of early-life characteristics, females with pediatric asthma had a higher proportion reporting poorer overall health up to 10 years old (25.4% vs 11.0%), had a higher proportion having plumper body size at 10 years old (16.2% vs 8.4%), had a higher proportion of parental smoking at birth (29.2% vs 22.4%), and had a higher proportion of lower birthweight (26.2% vs 15.7%), compared to non-asthmatic females.

**Table 2** shows the risk ratios (RRs) of the associations between pediatric asthma (diagnosed at 0-19 years of age) and adult PCOS (diagnosed at ≥20 years of age). In the crude and fully adjusted model, compared to non-asthmatics, pediatric asthma was significantly associated with adult PCOS (RR=1.73, 95% CI: 1.10-2.72 in the crude model; and RR=1.56, 95% CI: 1.02-2.41 in the adjusted model). In the restriction analysis by early-life risk factors, namely, body size at 10 years old and childhood health status up to 10 years old, the significance diminished among those who had about average and plumper body size, and those who reported excellent or good childhood health. Meanwhile, marginal significance was observed among those who reported poor or fair childhood health. Lastly, the statistical significance persisted among those with thinner body size at 10 years old (RR=2.00, 95% CI: 1.01-3.99 in the crude model; and RR=2.06, 95% CI: 1.08-3.93 in the adjusted model).

**Table 3** shows the risk ratios (RRs) of the associations between pediatric asthma (diagnosed at 0-19 years of age) and adult PCOS (diagnosed at ≥20 years of age), stratified by the age of adult PCOS diagnosis (20-25 years vs >25 years at PCOS diagnosis), in the main analysis and restricted by early-life risk factors, namely, body size at 10 years old and childhood health status up to 10 years old. In the young adult PCOS diagnosis stratum (diagnosed at 20-25 years), pediatric asthma was not associated with adult PCOS. On the contrary, in the older adult PCOS diagnosis stratum (diagnosed at >25 years) and after adjusting for confounding, we found that pediatric asthma had a significantly increased risk for adult PCOS (RR=2.06, 95% CI: 1.16-3.65). The statistical significance was still observed among those with thinner and about average body size at 10 years old in the older adult PCOS group even after adjusting for confounding factors (RR=2.74, 95% CI: 1.22-6.15 among thinner childhood body size category; and RR=3.40, 95% CI: 1.49-7.73 among about average body size category).

**Table 4** presents the risk ratios (RRs) of the associations between pediatric asthma (diagnosed at 0-19 years of age) and adult PCOS (diagnosed at ≥20 years of age), stratified by the age of asthma diagnosis (0-10 vs 11-19 years at asthma diagnosis), in main analysis and restricted by early-life risk factors, namely, body size at 10 years old and childhood health status up to 10 years old. We did not find any statistically significant associations in the main analysis for both strata. However, in the 0-10 years of age of asthma diagnosis and after adjusting for confounding, the significance was observed among those with poor or fair childhood health status (RR=2.54, 95% CI: 1.03-6.30). Meanwhile, in the 11-19 years of age of asthma diagnosis stratum and after adjusting for confounding factors, we found that those diagnosed with asthma at 11-19 years had a significantly increased risk of adult PCOS among those reported thinner childhood body size and those reported excellent or good childhood health status (RR=3.50, 95% CI: 1.38-8.43 among those reported thinner childhood body size, and RR=2.13, 95% CI: 1.01-4.50 among those reported excellent or good childhood health status).

## 4 Discussion

To our knowledge, this is the first population-based study evaluating the association between pediatric asthma diagnosed at 0-19 years and adult PCOS diagnosed at 20 years of age or above. We are able to demonstrate temporality as there is a long interval time between pediatric asthma and adult PCOS diagnoses in this analysis. In both crude and adjusted models and compared to non-asthmatic females, we found that pediatric asthma was significantly positively associated with adult PCOS (**Table 2**). Previous studies have found that PCOS was an independent risk factor for asthma among reproductive-aged females and suggested a strong correlation between PCOS and chronic systemic inflammation such as asthma (12–14, 31). However, our study provided a new perspective on how asthma and PCOS might be linked in the opposite direction. Our recent bi-directional study examining asthma and PCOS found a significant association between asthma diagnosed at <25 years and adult PCOS diagnosed at ≥25 years, independent of age and BMI (30). Female hormonal disturbance, metabolic syndrome, and obesity have been suggested as overlapping mechanisms that link asthma and PCOS (11–14, 31). A previous study has shown that obesity may worsen hormone dysregulation (25), and although there is no exact explanation linking asthma and metabolic syndrome, there were various known risk factors, including obesity or high BMI and dyslipidemia (44). Compared to non-asthmatics, those with pediatric asthma are known to be more susceptible to subsequent chronic diseases due to immune system impairment and persistent systemic inflammation (45, 46). In addition, compared to their healthy counterparts, pediatric asthmatics are known to have lower sympathetic nervous activity, and thereby a lower metabolic rate which may subsequently affect their vital biological process such as growth and reproduction (47–49). A previous study that linked pediatric asthma and reproductive health found pediatric asthma to be significantly associated with an earlier age at menarche (37). Therefore, we believe there are possible mechanisms involving biological factors in the association between pediatric asthma and adult PCOS since PCOS has been recognized as a chronic metabolic condition beyond a merely reproductive disorder (50).

We stratified by the age of PCOS diagnosis (**Table 3**) to better understand the association between pediatric asthma and adult PCOS phenotypes; young adult PCOS (diagnosed at 20-25 years), and older adult PCOS (diagnosed at >25 years). Compared to non-asthmatics, we found pediatric asthma was significantly associated with older adult PCOS. Most PCOS-related studies have involved adult populations (mean age > 25 years), however, PCOS can also be present in adolescence and young adulthood (aged ≤25 years) (51, 52). To our knowledge, there is no study on the association between pediatric asthma and any PCOS phenotypes (young adult PCOS or older adult PCOS) to compare to our study findings, however, several possible mechanisms may explain the observed findings. The expression of PCOS in early adulthood may differ from and does not necessarily resemble that of clinical and endocrinological features observed in later adulthood (52). Elevated sex hormone of adrenal androgen was observed in females with PCOS (8), and a previous study found that a greater decrease in adrenal androgen secretion happens between the ages of 20 to 25 years (53). In addition, inflammation is known to be a key factor in the pathology of asthma (2), and a previous study has shown that inflammation affects the level of female sex hormones (11). We believe possible mechanisms involving chronic inflammation and endocrinological features or sex hormones may explain the observed association between pediatric asthma and older adult PCOS phenotype in our study. Future studies to elucidate the exact mechanism of the association between pediatric asthma and older adult PCOS are warranted.

We further stratified the analysis by the age of asthma diagnosis (**Table 4**) to separate two distinct asthma phenotypes of childhood asthma (diagnosed at 0-10 years of age) and adolescent asthma (diagnosed at 11-19 years of age) and its association with adult PCOS. Childhood asthma (asthma diagnosed at 0-10 years) and adolescent asthma (asthma diagnosed at 11-19 years) were shown to be not significantly associated with adult PCOS in the crude and adjusted models. Asthma is known to be a uniquely diverse disorder with many clinical expressions throughout childhood and adolescence period (3). Childhood asthma and adolescent asthma are shown to be distinct in several ways, including their risk factors. The main risk factor for childhood asthma is a genetic predisposition (4), whereas the main risk factor for adolescent asthma is related to sex hormones (3). Our significant association between pediatric asthma and adult PCOS was only observed among those diagnosed with asthma at 11-19 years in the crude analysis, but the significant association disappeared after adjusting for confounding factors. Adolescence is a transitional stage of physical and psychological development, which is marked by the puberty period in which changes in reproductive hormones occur, and body weight gain following menarche is suggested to mediate the association between pediatric asthma and adult PCOS (54). Compared to healthier counterparts, pediatric asthmatics tend to have a lower sympathetic activity, hence a lower metabolic rate that may affect fat storage and may lead to overweight or obesity (47, 48). In addition, weight gain at puberty has been shown to be a significant risk factor for adult PCOS (52). The involvement of weight gain during puberty in the association between adolescent asthma and adult PCOS may be worth further investigation.

We also performed restriction analysis by body size at 10 years old and childhood health status up to 10 years old in all analyses (**Table 2**-**4**) to further explore the role of obesity in the association between pediatric asthma and adult PCOS since we could not rule out the possibility that childhood obesity and childhood health status may mediate the association between pediatric asthma and adult PCOS. Further analysis showed that childhood obesity or childhood health status alone was unlikely to explain the mechanism between pediatric asthma and adult PCOS, as the observed significance disappeared when the analysis was restricted to those with average or plumper body size at 10 years old, as well as to those with poorer/fair or excellent/good childhood health status up to 10 years old (**Table 2**). Childhood body size (obesity or overweight) may be related to pediatric asthma and adult PCOS through the following mechanisms. In addition to their lower metabolic rate as previously mentioned (47, 48), pediatric asthmatics are known to have poorer childhood health and may limit their physical activities due to their asthma conditions, hence, more susceptible to subsequent childhood overweight or obesity, compared to their healthy counterparts (37, 55). However, due to the design of this study, we could not further evaluate whether childhood obesity or childhood health status indeed mediated the association between pediatric asthma and adult PCOS.

### 4.1 Strengths and limitations

To our knowledge, our study is unique because it is the first population-based study examining the association between pediatric asthma and adult PCOS. We were able to assess the association with clear temporality and were able to control for relevant confounding factors. In addition, the large sample size in this study was suitable to perform stratification and/or restriction analysis to better assess and address the potential mediating effect on the association between pediatric asthma and adult PCOS. Epidemiological study of PCOS during different life stages is still limited and this study might guide future research.

Despite the strength of our study, we acknowledge some limitations. One major limitation pertains to the self-reported diagnosis of asthma and PCOS which might raise the concern about disease ascertainment accuracy. However, previous studies have shown self-reported asthma and PCOS diagnoses to be reliable (56–58). Self-reported age of asthma diagnosis was found to be accurate and had a low variability across categories of demographic and health-related characteristics (56), and self-reported PCOS was found to have high sensitivity in predicting PCOS (78%) compared to the sensitivity of PCOS diagnosis using the Rotterdam criteria (89%) (59). We have also adjusted for age in our adjusted analysis to further address the recall error for all self-reported variables. Furthermore, our study was also prone to misclassification, especially regarding childhood variables such as childhood body size, childhood health status, and parental smoking. However, we believe asthmatics and non-asthmatics in this study reported childhood variables in a similar fashion, resulting in non-differential misclassification. With regards to the convenience sampling design employed in our study, we have attempted to improve the representativeness of the sample by inviting the entire eligible population of the UAE to participate and operating multiple recruitment centers in different regions across the country to ensure ease of access. Another limitation is that we did not have information on the severity of asthma and PCOS to examine the associations involving disease severity (31). Our study may be prone to survivorship bias since we only included those who were alive at the questionnaire time; however, childhood mortality rates are low in the UAE and this is likely to have had a minimal effect on the findings (60). Our study also a had low sample size and statistical power in certain stratification analyses, such as in certain strata in the childhood body size and childhood health status (with N/A reflecting insufficient sample size). Lastly, our study may have been subjected to unmeasured confounding factors, including those factors with significant missing values such as parental education (40, 41) and birthweight (42, 43). However, our sensitivity analysis revealed similar results with or without the involvement of parental education and birthweight in the adjusted model (data not shown).

### 4.2 Conclusions

Our data demonstrated that pediatric asthma was an independent risk factor for adult PCOS. More targeted surveillance for those at risk of adult PCOS among pediatric asthmatics, may prevent or delay adult PCOS occurrence in this at-risk group. Future population-based studies with robust longitudinal designs aimed to elucidate the exact mechanism between pediatric asthma and PCOS are warranted.

## Institutional review board statement

The study and its procedures have been reviewed and approved by the Institutional Review Board at New York University Abu Dhabi, Dubai Health Authority, Ministry of Health and Prevention in the UAE, and Health Research and Technology Committee, reference number DOH/HQD/2020/516.

## Informed consent statement

Written consent was obtained from participants at the centers or by filling out an online consent form before data collection started.

## Data availability statement

The raw data supporting the conclusions of this article will be made available by the authors, without undue reservation.

## Ethics statement

The studies involving human participants were reviewed and approved by The study and its procedures have been reviewed and approved by the Institutional Review Board at New York University Abu Dhabi, Dubai Health Authority, Ministry of Health and Prevention in the UAE, and Health Research and Technology Committee, reference number DOH/HQD/2020/516. The patients/participants provided their written informed consent to participate in this study.

## Author contributions

Conceptualization, NJ, RA. Formal analysis, NJ. Data curation, NJ. Writing—original draft preparation, NJ. Writing—review and editing, AAb, AAh, AL-J, ASA., EA, FM, FA-M, FA, HA, JA, LW, MA, MK, MW, MA-H, MH-A, NO, OE-S, SS, SMS, TL, WA, YI, RA. Funding acquisition, RA All authors contributed to the article and approved the submitted version.
